# Prolonged central nervous system and respiratory depression in preterm neonates after exposure to brimonidine tartrate and timolol maleate ophthalmic drops

**DOI:** 10.3205/oc000152

**Published:** 2020-05-04

**Authors:** Corrina P. Azarcon, Darby E. Santiago

**Affiliations:** 1 Department of Ophthalmology and Visual Sciences, Philippine General Hospital, Manila, Philippines; 2 University of the Philippines College of Medicine, Manila, Philippines

**Keywords:** brimonidine tartrate, timolol maleate, central nervous system depression, apnea, preterm neonates

## Abstract

**Objective:** We report three cases of preterm neonates who presented with central nervous system (CNS) and respiratory depression after inadvertent exposure to brimonidine tartrate 0.2% and timolol maleate 0.5% fixed-combination ophthalmic drops.

**Case descriptions:** CNS and respiratory depression were observed in the three neonates within two hours of administration of brimonidine tartrate 0.2% and timolol maleate 0.5% eye drops. Respiratory support was initiated upon admission to the neonatal intensive care unit (NICU). The effects of the drug combination lasted for 24 to 48 hours.

**Conclusion:** This case series suggests that the drug combination of brimonidine tartrate and timolol maleate causes a prolonged depression of the central nervous and respiratory systems in preterm neonates.

## Introduction

Brimonidine tartrate 0.2% and timolol maleate 0.5% eye drops (Glubrim; Pharmtak Ophthalmics (I) Pvt. Ltd., Mumbai, India) is a fixed-combination ophthalmic drug preparation known to be effective in lowering intraocular pressure in patients with ocular hypertension and glaucoma [1], [2], [3]. The fixed combination of brimonidine tartate and timolol maleate achieves an improved control of intraocular pressure compared to monotherapy with either of the two components [1], [2], [3]. Eye irritation, dry eye, and allergic reaction were cited as the most common adverse effects among patients diagnosed with normal tension glaucoma aged 18 years old and above [3].

Brimonidine is a selective alpha-2 adrenergic agonist that acts by two mechanisms: via reduction of aqueous flow and via stimulation of uveoscleral outflow [3], [4]. Reduction of aqueous humor production is thought to be secondary to vasoconstriction of uveal blood vessels [5]. Increase in uveoscleral outflow is brought about by alpha-agonist induced prostaglandin synthesis, which causes ciliary muscle relaxation [5]. The lipophilic nature of the brimonidine permits rapid absorption through the cornea and transit through the blood-brain barrier, hence its potential to cause effects in the central nervous system (CNS) [6], [7], [8], [9]. CNS depression and apnea have been documented as adverse effects on neonates; its use in children less than two years of age is thus contraindicated [9]. Brimonidine that reaches the systemic circulation after drug absorption through the cornea undergoes rapid metabolism [10], [11]. Maximum plasma concentrations are seen 1 to 4 hours after administration; the half-life is about two hours [10], [11], [12]. Brimonidine undergoes extensive metabolism in the liver and is excreted in the urine [13].

Timolol is a beta-adrenergic blocker that binds beta-receptors on the ciliary epithelium, causing a decrease in aqueous humor production [3], [9]. Systemic beta-blockade occurs through the conjunctival, lacrimal, nasal, and gastrointestinal absorption of the drug after ocular administration [14]. Extensive systemic absorption may take place and cause effects on the cardiovascular, pulmonary, central nervous, and endocrine systems [14], [15], [16]. The half-life of timolol is 4 to 5 hours [17], [18]. Hepatic cytochrome P450 2D6 enzymes metabolize the drug into inactive metabolites that are excreted in the urine [14].

In this report, we discuss the case of three preterm neonates who were inadvertently given brimonidine tartrate 0.2% and timolol maleate 0.5% ophthalmic drops. This is the first case series describing the toxic effects of the fixed combination drug to neonates in the perinatal age group.

## Case descriptions

Three apparently well 1- to 2-day-old female preterm neonates delivered via spontaneous vaginal delivery were roomed-in and scheduled for retinopathy of prematurity screening. Table 1 (Tab. 1) displays the baseline characteristics of the subjects in this study. Brimonidine tartrate 0.2% and timolol maleate 0.5% eye drops were administered to the neonates instead of a usual mydriatic agent. Symptoms of CNS and respiratory depression were observed within 1 to 2 hours of administration warranting respiratory support and admission in the neonatal intensive care unit (NICU) for 24 to 48 hours.

### Case 1

Two drops of brimonidine tartrate 0.2% and timolol maleate 0.5% were administered on each eye of a 2-day-old female with physiologic jaundice. Bradycardia, bradypnea, and desaturation occurred persistently within one hour after exposure to the drug. Vigorous stimulation was done to interrupt these episodes. Nasal continuous positive airway support (NCPAP) was initiated at the NICU. Occurrence of bradycardia, apnea, and desaturation to less than 90% oxygen saturation while on NCPAP warranted switching of respiratory support to non-invasive positive pressure ventilation (NIPPV). Immediate improvement of vital signs was achieved after initiation of NIPPV. Phototherapy was administered for the jaundice. A thermal blanket was provided to manage episodes of hypothermia seen up to 8 hours after exposure to the drug. Weaning from NIPPV was started after 12 hours of its initiation. During this period, the patient already had spontaneous respirations, although hypopneic episodes were still observed up to 24 hours after the exposure. Throughout the first 24 hours after drug administration, the patient presented with poor activity; very minimal spontaneous movements were noted. On the next day, occasional movement of the extremities were already observed. At 36 hours post-exposure, the neonate was put on trial of room air. Forty-eight hours after exposure, the baby exhibited normal respiration with good activity, good suck, and good cry. The neonate was transferred back to the ward 72 hours after exposure and was discharged subsequently with no noted residual effects of the drug.

### Case 2

A 2-day-old female infant of a mother with gestational diabetes mellitus was inadvertently given 2 drops of brimonidine tartrate 0.2% and timolol maleate 0.5% drops per eye. The patient was seen drowsy 30 minutes after administration, but was consistently arousable to mild stimuli. Good muscle tone was maintained. Episodes of bradypnea occurring about 2 to 3 times every 10 minutes were interrupted through tactile stimulation. One hour after exposure, muscle tone was markedly decreased. The neonate became persistently bradypneic and was less responsive to tactile stimuli. The patient was transferred to the NICU where respiratory support via NCPAP was provided. Within the first few hours after administration, vital signs remained stable; the neonate was asleep but arousable to moderate stimuli. Six hours after exposure, the baby had good pulses; respiratory rate was 30 to 40 cycles per minute, and heart rate was 140 to 150. Spontaneous movements of all extremities were observed. A warm blanket was wrapped around the neonate for occasional episodes of hypothermia. Bradypneic episodes were noted up to 9 hours after exposure. Weaning from respiratory support was started after 15 hours of exposure. Room air was tolerated 24 hours post-exposure. Thirty-six hours after administration of the drug, the neonate was assessed to have good activity and good suck. The baby was allowed to stay at the ward with the mother 48 hours after exposure. The neonate was discharged from the ward on the next day with no residual effects of the eye drops.

### Case 3

An apparently well 1-day-old neonate was accidentally given two drops of brimonidine tartrate 0.2% and timolol maleate 0.5% fixed combination ophthalmic drops on one eye. Thirty minutes to 1 hour after exposure, the neonate was seen awake and breastfeeding. Decreased sensorium and bradypnea was observed at about 1 to 2 hours after administration of the combination drug. The neonate was admitted to the neonatal ICU where NCPAP was administered. Bradypneic and bradycardic episodes were recorded up to 16 hours after drug administration. A thermal blanket was provided for hypothermic episodes that occurred up to 24 hours post-exposure. Weaning was begun as soon as 10 hours after initiation of respiratory support. Room air was tolerated 24 hours post-exposure. The baby was assessed to have good activity and good suck at 36 hours after drug administration. The neonate was discharged from NICU and was brought back to the regular ward at the 48^th^ hour post-exposure. The patient was discharged from the ward a couple of days after with no residual effects from the incident.

## Discussion

Brimonidine is an imidazole derivative that acts as a selective alpha-2 agonist [9], [19]. It is structurally similar to clonidine, another alpha-2 agonist drug that has a well-documented toxicity profile in the pediatric population [19], [20]. Clonidine binds to alpha-2 adrenergic receptors and imidazole receptors on the rostral ventrolateral medulla [19]. The binding of the drug to imidazole receptors results in central lowering of blood pressure, hence its pharmacologic use in the control of hypertension and tachycardia [19]. Decrease in pupillary diameter, sensorium, respiratory drive, heart rate, blood pressure, and temperature have been observed among exposed children of less than 2 years of age within 30 minutes to 1 hour of exposure [19]. Effects are expected to last up to 24 hours [19]. The chemical similarity of brimonidine to clonidine accounts for its similar effects on patients [19]. However, brimonidine is expected to have less systemic toxicity compared to clonidine due to its lower lipid solubility [19], [20].

Literature shows plentiful evidence on the toxicity of brimonidine in the pediatric population [6], [7], [20], [21], [22], [23]. A study reported a case of an 11-day-old neonate with persistent hyperplastic primary vasculature who was given topical brimonidine and echothiophate iodide for post-surgical elevation of intraocular pressure [20]. The neonate became lethargic, apneic, hypotonic, and pale within 10 minutes after being given 1 drop of each of the drugs per eye. Similar observations were noted when brimonidine alone was applied; upon discontinuation, the patient became asymptomatic. The same study reported of a 5-month-old infant that presented with lethargy 30 minutes after being given 1 drop of brimonidine per eye. Effects resolved completely after 2.5 hours [20]. A retrospective study investigated the frequency and trends in brimonidine tartrate pediatric poisoning and the use of naloxone as an antidote using data from the American Association of Poison Control Centers’ Toxic Exposure Surveillance System database and the US Food and Drug Administration’s Medwatch Adverse Events Reporting System from 1997 to 2005. It was found that the peak age of exposure to brimonidine was 2 years old, and a significant proportion of the pediatric poisoning cases resulted from accidental ingestion. Drowsiness was the most common symptom reported; other symptoms recorded were ataxia, pallor, irritability, hypotension, bradycardia, miosis, and respiratory depression. More than 10 percent of poison control centers recommended the use of naloxone in emergency cases, but its role remains unclear [21].

Studies on the toxicity of ophthalmic timolol are also available [15], [16], [24], [25], [26], [27]. Timolol is a beta-adrenergic blocker that can cause a neural depressant effect by crossing the blood-brain barrier and binding to receptors that control behavior-associated information flow and beta-adrenergic tone [15]. A randomized crossover study has found that 80% of 0.5% timolol ophthalmic eye drops are absorbed systemically [24], [25]. Among adults, CNS effects such as memory loss, confusion, depression, hallucination, and headache have been identified as the most common systemic side effect from ocular administration of timolol maleate, with an incidence of about 10 percent [15]. A portion of the reports are specific to infants [25], [28], [29], [30]. A 2-week-old preterm neonate diagnosed with congenital glaucoma presented with apneic spells lasting for 30 seconds after treatment with timolol maleate and cyclotherapy [28]. The spells were absent when cyclotherapy was used alone [28]. In another study, apnea was observed after post-operative administration of timolol in neonates and young infants [29]. Cardiorespiratory depression was observed after accidental nasal administration of topical timolol in an 8-month-old male who was taking propranolol as a maintenance drug [30]. The off-label use of topical timolol for infantile hemangiomas has also been found to cause bradycardia among young and preterm infants [25].

Literature search only yielded 2 reports on the combined toxicity of brimonidine tartrate and timolol maleate on infants [31], [32]. A study discussed 2 cases of infants exposed to anti-glaucoma drugs [31]. The first infant was a 5-month-old female who was given topical brimonidine and brinzolamide upon diagnosis of congenital glaucoma. CNS and respiratory depression were attributed to brimonidine. The infant recovered completely after 9 hours of intensive monitoring. Similar but more profound effects were observed 30 minutes after a 3-month-old female was given topical brimonidine, brinzolamide, and timolol. These signs were accompanied by bronchoconstriction and signs of cardiogenic shock. Intravenous hydration, atropine, and dopamine were administered. Salbutamol was given for broncho-obstruction. Recovery was complete after more than 12 hours. The report showed that ophthalmic administration of timolol with brimonidine added a risk of bronchoconstriction and cardiac depression on top of CNS depressive effects. Mungan et al. [32] reported of 2 infants who were given brimonidine along with beta-blockers. The first infant was a 3-week-old neonate who became apneic, bradycardic, hypotensive, and hypothermic within minutes after administration of 0.2% brimonidine and 0.25% timolol eye drops. Symptoms necessitated intubation but the neonate recovered and was sent home the next day. The second infant was an 8-week-old girl given 0.5% topical betaxolol, 2% dorzolamide, and 0.2% brimonidine. Bradycardia, hypoventilation, and hypoxemia occurred but the infant was consistently responsive to stimulation. Recovery was complete within 24 hours. It was hypothesized that beta-blockers act synergistically with brimonidine in stimulating peripheral adrenergic receptors.

Previous reports on the exposure of infants to brimonidine or timolol eye drops have shown that the adverse effects of these drugs, taken individually, are expected to last for less than 24 hours. In this case series, the effects of the fixed combination of brimonidine tartrate and timolol maleate on the neonates lasted for 24 hours up to 48 hours after exposure. This duration is at least twice longer than what has been observed for older infants who were given either or both the drug components. The vulnerability observed among preterm neonates towards eye drops may be attributed to the following:

1. immature blood-brain barrier,
2. deficiency of certain cytochrome P450 enzymes,
3. lack of pediatric delivery devices that allow delivery of weight-appropriate doses, and
4. poorly controlled drug administration into the eye [33].

In addition, doses of eye drops are not adjusted according to weight, thus making children more vulnerable to toxicity due to high dose-weight ratios [30]. In this case series we further note that the toxic effects of the drugs were more marked in the patient in Case 1. Compared to the patients from the two other cases, the patient in Case 1 had the lowest weight and Ballard’s score, which signify a greater degree of physical immaturity. The presence of jaundice in this neonate also denotes relative insufficiency of metabolic enzymes. This was a predicted outcome since both brimonidine and timolol are metabolized hepatically. A potential synergistic effect of the two drugs is a possible explanation for the prolonged CNS and respiratory depression observed in the neonates in this case series.

The neonatal toxicity of brimonidine tartrate and timolol maleate eye drops should be managed in the hospital where adequate respiratory and cardiovascular support can be provided. Although the effects of the two components are temporary, supportive therapy should be provided to ensure continuous metabolic function of the neonate’s organ systems. For patients with marked beta-blockade, intravenous atropine may be administered for severe bradycardia and hypotension; intravenous glucagon may be given for cardiogenic shock [30]. Respiratory depression may be managed by tactile stimulation for very mild cases and by NCPAP or NIPPV, as seen in this study. Intubation may be warranted for more severe cases.

## Conclusion

The adverse effects of ophthalmic preparations of brimonidine and timolol on the pediatric population have been documented in previous literature. We report three cases of 1- to 2-day-old neonates who presented with prolonged central nervous system and respiratory depression upon inadvertent administration of a fixed combination of brimonidine and timolol. The duration of symptoms seen in the preterm neonates in this case series is at least twice longer compared to the duration seen in previous cases of pediatric exposure to individual preparations of the two drugs. The increased toxicity in the perinatal period may be secondary to a high eye drop dosage to neonatal weight ratio, immature liver enzymes, and immature blood brain barrier [30], [33]. The presence of co-morbid conditions is also a plausible factor in the prolongation of the systemic side effects. A possible synergistic toxic effect of brimonidine and timolol cannot be ruled out. Respiratory support may be warranted for a longer period in preterm neonates. No long-term sequelae are reported in literature.

This study has shown that the toxicity of the combination drug brimonidine tartate 0.2% and timolol maleate 0.5% eye drops in neonates is life-threatening, and it supports that the use of the drug in children less than 2 years of age is contraindicated. Extra caution should be observed when administering eye drops in neonates due to the increased toxicity of eye drops in this age group.

## Notes

### Competing interests

The authors declare that they have no competing interests.

### Acknowledgement

The authors would like to thank the Philippine General Hospital Department of Pediatrics for providing the data on the course of the patients.

## Figures and Tables

**Table 1:**
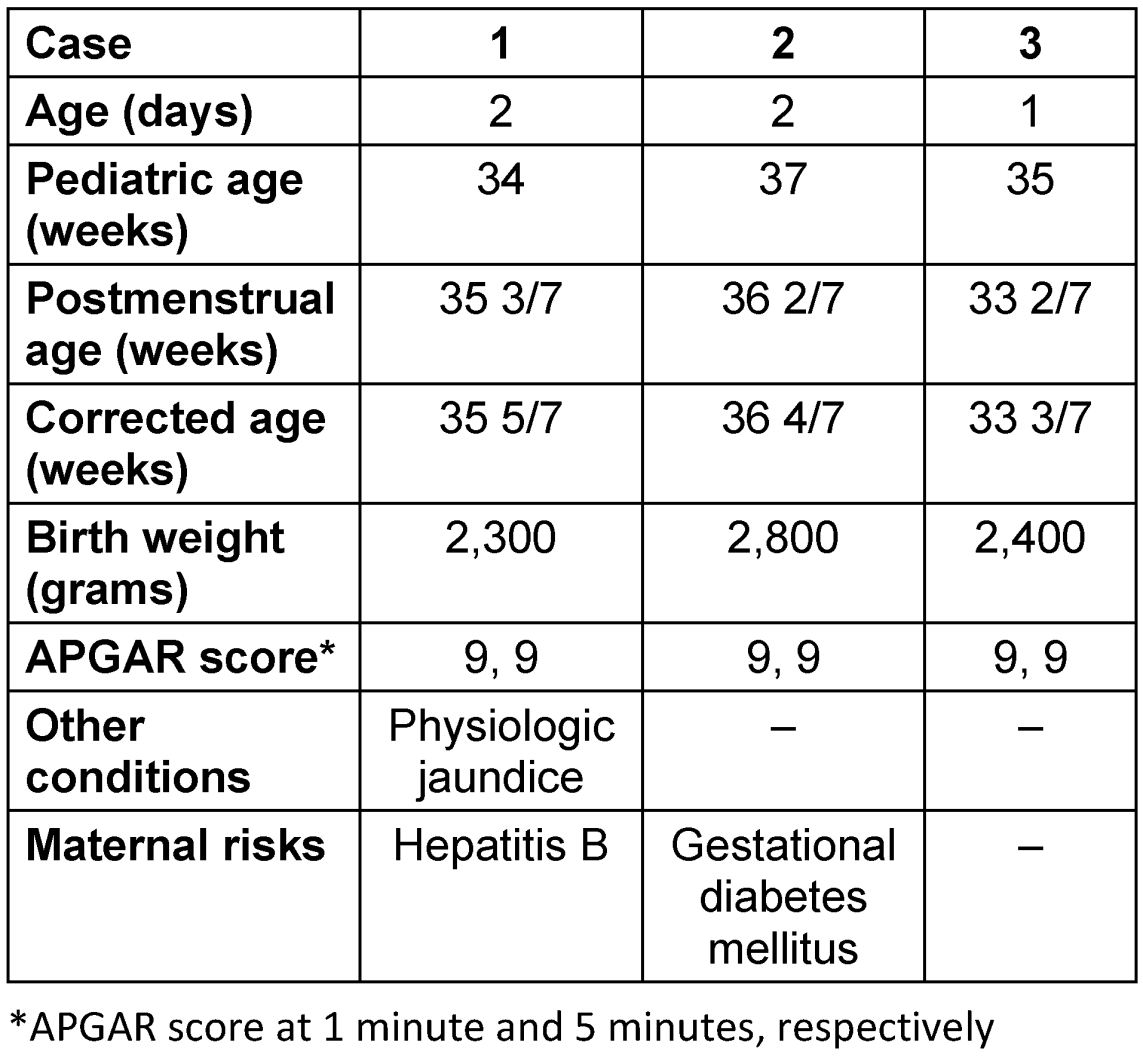
Baseline characteristics of preterm neonates exposed to brimonidiine tartrate 0.2% and timolol maleate 0.5% ophthalmic drops
